# Understanding the dimensions of mental labor: the invisible load of Italian mothers

**DOI:** 10.3389/fsoc.2025.1683261

**Published:** 2026-01-29

**Authors:** Elena Vettoretto, Alessandra Minello, Livia Elisa Ortensi, Francesca Tosi

**Affiliations:** 1 Department of Statistical Sciences, University of Padova, Padova, Italy; 2 Department of Political Science, Law and International Studies, University of Padova, Padova, Italy; 3 Alma Mater Studiorum – University of Bologna, Department of Statistical Sciences “Paolo Fortunati”, Bologna, Italy

**Keywords:** domestic labor, family, gender inequality, Italy, mental load

## Abstract

**Introduction:**

Mental labor—encompassing the planning, anticipating, and emotional monitoring required to manage family life—represents an invisible yet unequally distributed component of unpaid domestic work. Despite growing attention, little is known about how mental labor operates within families and which factors shape its distribution, particularly in the Italian context.

**Methods:**

The study draws on survey data from 2,309 Italian mothers of one child and employs a multidimensional scale capturing cognitive, managerial, and emotional dimensions of mental labor. We analyze both the total amount of mental labor reported by mothers (individual sphere) and the perceived gap between their own and their partner’s contribution (relational sphere). Ordinary Least Squares (OLS) regression models are used to identify individual and relational predictors of mental labor intensity and perceived inequality.

**Results:**

Findings reveal that mothers carry a substantial mental load, with the cognitive dimension being particularly pronounced. Individual-level characteristics, such as gender role attitudes and employment status, predict the overall intensity of mental labor. Relational dynamics—especially the partner’s working hours and degree of practical support—are more strongly associated with the perceived gap in contribution between partners.

**Discussion:**

These results highlight the persistence of gendered divisions in the organization of unpaid care in Italy. The unequal mental load borne by mothers underscores the need to address both individual and relational determinants of family work distribution, with implications for well-being, gender equity, and family policy.

## 1 Introduction

Despite significant progress in women’s participation in education and the labor market, the domestic sphere continues to be shaped by persistent gender inequalities. The unequal distribution of unpaid domestic labor within couples has been extensively studied in the sociological literature: research consistently demonstrates that, despite their participation in the paid labor force, women continue to shoulder a disproportionate share of unpaid domestic and caregiving tasks (Bianchi and Milkie, 2010; Perry-Jenkins and Gerstel, 2020; Goldin, 2021). Most research has focused on visible aspects, such as household chores and physical tasks, often measured in terms of time spent (e.g., Balbo et al., 2024; Gauthier et al., 2004; Sandberg and Hofferth, 2001). Unpaid domestic work takes visible forms, such as cleaning, cooking, and childcare (Craig and Mullan, 2010), but also has less tangible mental features. This “mental workload” reflects the internal, often invisible labor in anticipating needs, organizing routines, worrying about the well-being of others, and managing family logistics and relationships (Daminger, 2019; Wayne et al., 2023). Although the concept of mental labor has gained traction in both public and academic discourse, it remains relatively unexplored in empirical research. Earlier studies of unpaid domestic work focused primarily on time use and the gendered division of physical tasks (Bianchi et al., 2000; Craig and Mullan, 2010), often overlooking the emotional and cognitive effort required to sustain household functioning. More recently, however, a growing body of literature has sought to illuminate this invisible labor—variously described as mental load, cognitive labor, or invisible family work—which encompasses remembering, planning, monitoring, and bearing responsibility for others (Hochschild and Machung, 1989; Walzer, 1996; Daminger, 2019; Weeks et al., 2025).

These tasks are demanding not only because of their complexity but also due to their continuous, boundaryless nature (Dean et al., 2021). Many women, particularly those in heterosexual couples, report feeling “always on,” mentally preoccupied even during paid work or leisure time (Walzer, 1998). High levels of emotional mental load have been linked to emotional exhaustion, sleep disturbances, work–family conflict, and lower job performance for women (Wayne et al., 2023; Haupt and Gelbgiser, 2023; Callaghan et al., 2024). These gendered effects of mental workload are consistently observed even in dual-earner or ostensibly egalitarian households (Offer, 2014; Ciciolla and Luthar, 2019), with far-reaching consequences for gender equality, individual well-being, and work-family balance.

Despite these important findings, several knowledge gaps remain. Most empirical studies have been conducted in North American contexts, with limited attention to countries like Italy, where strong familialism and enduring gender norms may intensify mental workload inequalities. Moreover, much of the existing research does not disaggregate mental labor into its distinct components (Wayne et al., 2023) – cognitive, managerial, and emotional—nor does it systematically explore variation across socio-demographic or relational factors. A further conceptual gap lies in the distinction between individual and relational spheres of mental workload. The individual one captures a woman’s subjective perception of her own cognitive, managerial, and emotional effort in managing family responsibilities—reflecting an internalized sense of responsibility, regardless of the partner’s contribution. The relational sphere, by contrast, refers to the perceived imbalance between the mental labor performed by oneself and one’s partner. This aspect highlights perceptions of fairness and the dynamics of inequality within heterosexual couples.

Measurement also presents a limitation. Earlier research has primarily relied on qualitative interviews or *ad hoc* inventories (Lee and Waite, 2005), which constrains comparability and generalizability. New evidence limited to the Italian North-Eastern region Emilia-Romagna, shows that women disproportionately bear both the cognitive and emotional dimensions of mental load, with organizational responsibilities closely linked to the actual execution of domestic and childcare tasks and a marked spillover into paid work, especially among employed and college-educated women (Barigozzi et al., 2025). A recent advancement is the Invisible Family Load Scale developed by Wayne et al. (2023), a nine-item instrument that captures the cognitive, emotional, and managerial facets of mental labor, which can serve as a starting point for measurements in specific cultural and contextual groups.

This study addresses these gaps by offering a multidimensional and empirically grounded analysis of mental labor in the Italian context. We assess Italian mothers’ mental workload across its cognitive, managerial, and emotional dimensions, distinguishing between individual and relational aspects, and controlling for maternal characteristics (i.e., education, employment, and gender attitudes) and relational level dynamics (such as partner’s employment status, and division of household tasks). In doing so, the paper contributes to a more comprehensive understanding of gendered unpaid labor in Italy, highlighting its invisible yet pervasive forms that undermine women’s well-being and impede progress toward gender equality. More specifically, in this study, we address three main research questions. First, we ask what factors shape the overall extent of mothers’ mental labor in Italy, paying particular attention to both individual characteristics and relational dynamics within couples (RQ1). Second, we investigate which individual and relational aspects explain the gap between mothers’ reported mental labor and the load they attribute to their partners (RQ2). Finally, we disaggregate mental labor into its cognitive, managerial, and emotional dimensions, exploring whether different aspects are associated with diverse responsibilities across these dimensions (RQ3).

The Italian context is particularly relevant for studying mental labor, as it already displays high levels of gender inequality in the more visible components of unpaid domestic work, including housework, childcare, and elder care (Dotti Sani, 2018; Mencarini and Tanturri, 2004).

The paper is structured as follows: Section 2 reviews the theoretical and empirical literature on mental workload. Section 3 discusses the research questions based on the identified knowledge gaps. Section 4 describes the data and methods used to investigate mothers’ mental workload in Italy. Section 5 reports the results. Section 6 concludes.

## 2 Mental labor as a complex issue

The unequal division of unpaid domestic labor has been extensively documented in sociological research, concentrating on its visible and measurable components, primarily the number of hours dedicated to tasks such as cleaning, cooking, and childcare (Craig and Mullan, 2010). This approach, grounded in time-use surveys, has significantly advanced our understanding of gender inequalities in household labor. Still, it has left out a critical dimension: the invisible labor required to coordinate, manage, and emotionally sustain family life. A growing body of research has sought to fill this conceptual gap by introducing the notion of mental labor to describe the internal, often unrecognized effort that makes the visible aspects of domestic life possible (Hochschild and Machung, 1989; Daminger, 2019). When women enter the labor market, if men do not increase their share of household work accordingly, women experience a “double burden” of paid and unpaid work (Hochschild and Machung, 1989; Oakley, 1974). Mental labor is less visible than housework or childcare, yet it may be even more persistently gendered. From a gender socialization perspective, following Social Role theory, women are taught from an early age to assume caregiving and relational responsibilities, which may predispose them to carry more of the mental load in adulthood (Eagly and Wood, 2016). At the same time, women may take on most of the mental load as a way of “doing gender” (West and Zimmerman, 1987), continuously enacting and reproducing gender roles through everyday practices. Finally, time availability approaches suggest that, while individuals engaged in full-time employment should in theory contribute less to unpaid domestic work, empirical evidence shows that full-time working mothers continue to bear much of the responsibility for managing and coordinating family life (Craig and Mullan, 2010; Goldscheider et al., 2015). The “gender revolution” (England, 2010), which entails the equal participation of men and women in caregiving, is still incomplete. Taken together, these perspectives indicate that mental labor is not simply an extension of visible domestic tasks, but a distinct dimension of household inequality that remains unequally distributed even when paid and unpaid labor appear more balanced.

The concept of mental load also builds on earlier feminist critiques of the domestic division of labor, particularly the notion of the “second shift” (Hochschild and Machung, 1989), by clarifying how gendered expectations extend beyond physical tasks to the mental and emotional responsibility for the household. In this perspective, mental load functions as a mechanism through which gender inequalities are reproduced: women are often expected to be “the family managers,” the ones who remember birthdays, schedule doctors’ appointments, or sense and manage the emotional needs of children and partners, even when these tasks are not explicitly assigned.

Empirical research consistently confirms that mental labor is disproportionately carried by women, even in dual-earner households and among couples who espouse egalitarian values (Weeks et al., 2025). In a qualitative study, Daminger (2019) finds that women overwhelmingly take on the anticipatory and coordination components of household work—particularly those that require identifying needs and ensuring they are addressed—while men tend to be more involved in the execution of delegated tasks. Similarly, using experience sampling methodology, Offer (2014) finds that mothers engage in more cross-domain and family-related mental labor than fathers, and that this burden negatively affects their emotional well-being. In contrast, fathers’ levels of mental labor appear unrelated to their well-being. Lee and Waite (2005) report that both husbands and wives spend approximately 2–3 h per week thinking about household responsibilities, but that wives spend, on average, 1 h more. Meier et al. (2006) find that fathers experience greater marital satisfaction when they are involved in direct childcare but not in its cognitive management, whereas mothers are more satisfied when they are emotionally engaged in childcare tasks but less responsible for general household planning. In qualitative interviews with elderly Swedish women, Forssen and Carlstedt (2008) highlight how caregiving entails both visible and invisible work—including constant preparedness and planning—which they describe as both mentally and physically strenuous. Ciciolla and Luthar (2019) show that greater maternal responsibility for children’s emotional development predicts lower life satisfaction and partner satisfaction, along with a pervasive sense of emotional depletion. In Italy, drawing on data from the TIMES Observatory, Barigozzi et al. (2025) show that women bear disproportionate responsibility for managing domestic organization, often without corresponding recognition or support. This mental load is associated with increased emotional fatigue, dissatisfaction with the division of labor, and frequent interference of family responsibilities during paid work hours—particularly among employed, highly educated women. Importantly, the perceived responsibility for managing household tasks appears more strongly tied to gender gaps in time use within couples than to the absolute time spent on specific chores, pointing to the relational, and at times conflictual, feature of mental labor.

Taken together, these studies demonstrate not only the unequal distribution of mental labor, but also its gendered psychological consequences. As mental labor tends to remain invisible and continuous, it can generate chronic emotional and cognitive strain, especially when unreciprocated or unacknowledged (Dean et al., 2021). It is thus unsurprising that a growing body of research has sought to explore its consequences for well-being, such as in Offer (2014), where the mental labor women perform in managing family life is found to contribute to emotional fatigue and stress, while having little to no effect on men. Haupt and Gelbgiser (2023) extend these findings, showing that women who carry the bulk of cognitive responsibility within the household are significantly more likely to report emotional depletion and fatigue. In contrast, men’s well-being is largely unaffected by the share of cognitive labor they report performing, even when they claim to do as much or more than their partner. These results point to an important asymmetry: the perceived weight of mental labor appears to be more psychologically burdensome for women than for men.

Importantly, these burdens are not merely psychological. The cumulative mental load experienced by women—especially when paired with paid employment—may also hinder their professional trajectories (Haupt and Gelbgiser, 2023). In this sense, mental labor contributes not only to domestic inequality but to broader patterns of gender inequality in the public sphere as well.

Given the individual and the relational gendered nature of mental labor, it becomes crucial to investigate what drives its magnitude and the unequal distribution within couples. Despite growing interest in the topic, empirical research has yet to systematically examine its predictors. As a result, we draw on the well-established literature on the division of unpaid domestic work to identify plausible explanatory factors, distinguishing between individual-level determinants, referring to the respondent’s own characteristics and attitudes, and relational-level determinants, which capture features of her relational context, such as the partner’s employment status or the availability of childcare support within the family.

On the individual level, education, for instance, has been shown to influence bargaining power and awareness of gender inequalities in the domestic sphere (Brines, 1994; Bianchi et al., 2000), potentially affecting how much invisible labor a woman undertakes. Employment status contributes via the logic of time availability: those engaged in full-time paid work have less discretionary time and are theoretically less involved in unpaid domestic tasks (Coverman, 1985), yet studies reveal that full-time working mothers still bear most management responsibilities (Craig and Mullan, 2010), suggesting that time alone does not explain mental burden. Marital status may structure internal expectations: cohabiting couples have been shown to divide household tasks more evenly than married ones (Davis et al., 2007), possibly reflecting less traditional gender framing. Furthermore, following social role theory (Eagly and Wood, 2016), we include gender role attitudes, as individuals with more egalitarian gender ideologies are more likely to engage in balanced domestic arrangements (Aassve et al., 2014; Grunow et al., 2012), which may also extend to the cognitive and emotional dimensions of household labor.

Relational aspects, in turn, refer to the characteristics of the partner and the overall dynamics within the couple. Even when execution is shared, women frequently retain the role of “household manager,” anticipating, organizing, and delegating tasks (Daminger, 2019). Partner’s employment status is a critical structural variable: full-time employed partners may have limited availability for mental labor, whereas part-time or flexible arrangements may facilitate greater involvement. Furthermore, practical help with household tasks provided by partners (as reported by mothers) serves as an indicator of perceived day-to-day support and may signal shared cognitive and emotional responsibility, potentially reducing the mental labor borne by mothers. Based on these insights, we formulate the following research questions:

RQ1. What are the key determinants of domestic mental labor among mothers in Italy? In particular, which individual and relational aspects predict higher mental labor among mothers?

RQ2. Which individual and relational aspects explain the gap between mothers’ reported mental labor and the load they attribute to their partners?

Recent theoretical developments have framed mental labor as a multidimensional construct, comprising at least three distinct but interrelated components (Wayne et al., 2023): (i) cognitive labor, which includes remembering, monitoring, anticipating, and mentally tracking the needs and schedules of household members; (ii) managerial labor, which refers to organizing, planning, delegating, and ensuring the execution of domestic activities; and (iii) emotional labor, which entails worrying, empathizing, providing emotional support, and maintaining the well-being of others. These dimensions reflect the complexity and embeddedness of mental labor in everyday life. They also highlight its continuity across activities and settings: mental labor does not stop when one leaves the home or completes a physical task. Instead, it occupies mental bandwidth, often concurrently with paid work or rest.

Wayne et al. (2023) offer further insights by disaggregating mental labor into its components. They state that while managerial and cognitive labor can, in some cases, be associated with positive outcomes—such as family-work enrichment, competence, and satisfaction – emotional labor consistently functions as a hindrance stressor. Characterized by chronic worry, responsibility for others’ emotional states, and anticipatory concern, they link emotional mental labor to emotional exhaustion, sleep disruption, work–family conflict, and reduced job performance.

Building on these insights, we respond to our last research question:

RQ3. How is mental labor distributed across its cognitive, emotional, and managerial dimensions? Which individual and relational aspects are associated with cognitive, emotional, and managerial higher mental labor among mothers?

## 3 Data and methods

Our analysis is based on data collected as part of the FORTIES (Fertility Over ForTIES) project, a web-based survey (CAWI—Computer Assisted Web Interview) conducted in Italy in Autumn 2024. The survey targets approximately 3,200 mothers aged 20–45 with one or two children, and the respondents were recruited through both an online panel and social media advertisements. Although the data are not based on a probabilistic sample, they approximate the Italian population of mothers aged 20–45, as they are proportional in terms of age and geographical distribution.

The broader aim of the FORTIES survey is to investigate fertility trajectories and experiences of motherhood, particularly in later life. However, the survey also includes a dedicated module on the division of household labor and the associated mental labor, making it an important and innovative resource for analyzing domestic gender dynamics beyond fertility.

Our analytical sample consists of mothers aged 20–45 who are currently in a heterosexual relationship and have one child, yielding a total of 2,309 valid observations. We focus on mothers of one child to ensure greater comparability across respondents in terms of caregiving demands and household complexity. This restriction allows us to isolate mental labor dynamics in a relatively uniform phase of the life course, avoiding the additional variability introduced by larger families, where domestic coordination and emotional demands are likely to differ significantly. Additionally, to assess the robustness of our results, we ran the analyses on the full original FORTIES sample, including mothers with more than one child, adding parity as a control variable (see Table A4 in the Appendix). The findings are consistent with our main analyses, thereby supporting the robustness of our conclusions. Furthermore, sampling weights were not applied because our analyses focus on the estimation of relationships among variables. Preliminary descriptive checks suggested only limited divergence between the sample and the broader population of mothers on key sociodemographic indicators, making unweighted analyses appropriate for our analytic goals. The study’s aim is to capture patterns of mental labor among Italian mothers rather than to provide population estimates.

### 3.1 Variables

To capture mental labor, the study relies on a 7-item scale inspired by a newly developed multidimensional scale (Wayne et al., 2023), designed to measure three core dimensions: (i) cognitive labor: remembering, monitoring, and thinking about tasks that need to be done; (ii) managerial labor: planning, organizing, and delegating tasks within the household; and (iii) emotional labor: experiencing stress, worry, and emotional responsibility for family members’ wellbeing. The survey instrument included seven items—one cognitive, three managerial, and three emotional—thus omitting two cognitive items from the original 9-item scale (Wayne et al., 2023). The overall mental labor index demonstrates good internal consistency (Cronbach’s *α* = 0.75), with *α* = 0.77 for the managerial and *α* = 0.86 for the emotional labor subdimensions.

Respondents assess these dimensions based on their current life stage. They are asked to indicate how frequently they engage in various mental labor activities, using a scale from 1 (“never”) to 5 (“always”).^1^ In addition, they are asked to report, based on their perception, the extent to which their partner is involved in the same activities. This dual-perspective approach enables us to examine not only the distribution of mental labor (individual aspect) but also the perceived gap between partners (relational aspect), offering insights into whether mental workload is experienced as a shared responsibility or a unilateral burden.

From this scale, we derive five dependent variables. First, total mental labor is computed as a weighted mean of the seven items. The single cognitive item receives a weight of one-third, while each of the remaining six items—three managerial and three emotional—receives a weight of one-ninth. This weighting scheme ensures that the three dimensions (cognitive, managerial, and emotional) contribute equally to the total index. Second, to measure the mental labor gap between mothers and their partners, we compute the difference between the mother’s average score and the perceived score of her partner. Third, cognitive labor is captured by a single item, while managerial and emotional labor are measured as the mean of their respective items.

These five variables serve as the outcome measures in our empirical analysis, which aims to address the three research questions outlined above through a series of OLS regression models using the five dependent variables: total mental labor (RQ1); the gap between mother’s and perceived partner’s mental labor (RQ2); and the three dimensions—cognitive, managerial, and emotional (RQ3).

Table 1 provides a detailed overview of the analytical sample: On the individual side, the average age of respondents is 37.18 years, with a sample ranging from 20 to 46 years. In terms of education, 63% of the mothers hold a tertiary degree, suggesting a highly educated sample. Employment status varies: 49% of the women are employed full-time, while 26% work part-time and 25% are either in flexible arrangements or are currently unemployed. We also consider the availability of help in child-rearing, a potentially important factor in shaping mental workload. The majority of mothers report receiving help from their partner (72%), while 26% rely on grandparents or friends, and only 3% report having no support at all. In addition, we include a measure of gender role attitudes, based on a seven-item scale from the ISSP (2022), and summarized into an index calculated as the arithmetic mean of the items included in the module, capturing individual views on the appropriate division of roles within the household. On average, mothers score 3.72 out of 5, indicating a moderately egalitarian orientation.

**Table 1:** Descriptive statistics (*N* = 2,309).

Variables	Mean/Prop.	Freq.	SD	Min.	Max.	IQR	Median
Total mental labor	3.82		0.67	1	5	1	3.88
Gap total mental labor	0.54		0.91	-3	4	1	0.33
Emotional labor	3.21		1.10	1	5	1.67	3.00
Managerial labor	3.96		0.77	1	5	1.33	4.00
Cognitive labor	4.30		0.92	1	5	1	5.00
Help in childcare							
Partner	0.72	1,657					
Grandparents and/or friends	0.26	589					
Nobody	0.03	63					
Gender role attitudes	3.72		0.77	1	5	1.14	3.71
Education: tertiary	0.63						
Mother employment status							
Full time	0.49	1,120					
Part-time	0.26	602					
Flexible or unemployed	0.25	587					
Mother age	37.18		5.88	20	46	8	39
Marital status							
Married	0.54	1,247					
Not married	0.46	1,062					
Partner employment status							
Full Time	0.86	1981					
Part-time	0.09	208					
Flexible or unemployed	0.05	120					
First child age	2.65		2.51	0	15	3	2
First child health							
Not good	0.10	224					
Good	0.25	588					
Very good	0.65	1,497					
Geographical area							
North–West	0.29	667					
North–East	0.23	527					
Central	0.20	471					
South and islands	0.28	644					
Recruitment: social	0.45						

Relational characteristics also play a central role. Just over half of the sample is married (54%), while 46% are in non-marital relationships. We include the employment status of the partner: a categorical variable assuming value 0 if the partner works full-time, 1 if the partner works part-time, and 2 if the partner has a flexible job or is unemployed. We include it as a structural proxy for their time availability and potential engagement in household labor: 86% of partners work full-time, 9% part-time, and 5% are in flexible or non-standard work arrangements. Child-related factors are also considered. The mean age of the first child is 2.65 years, and approximately 10% of mothers report that their child’s health at birth was not good, with the remaining reporting either good (25%) or very good (65%) health. These conditions may affect the intensity and emotional dimension of mental labor, especially when children are younger or have special care needs.

Finally, the sample is geographically well distributed across Italy: 29% of respondents reside in the North-West, 23% in the North-East, 20% in the Centre, and 28% in the South and Islands. This geographic spread reflects broader socio-economic and cultural differences across Italian regions, which are likely to intersect with family dynamics and gender norms. We also control for recruitment channel, with 45% of respondents recruited via social media and the rest from an online structured panel of respondents to account for possible variation in sample composition due to different outreach methods.

## 4 Results

**Figure 1:**
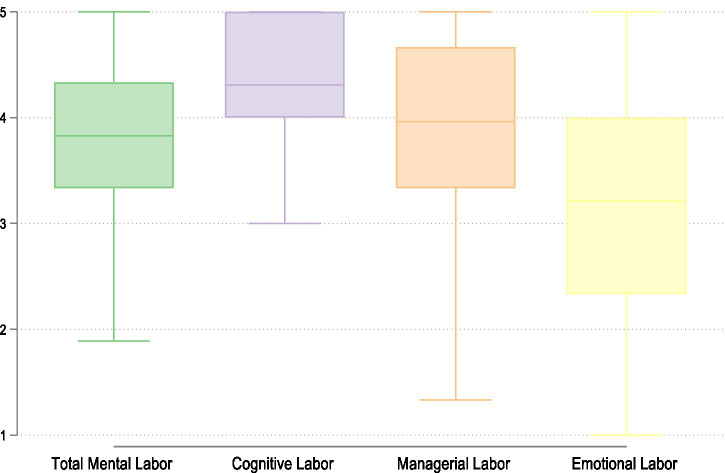
Perceived own total mental labor, cognitive labor, managerial labor, and emotional labor reported by Italian mothers of one child. Source: Own elaborations on FORTIES data (2024).

Figure 1 shows the levels of self-attributed mental labor, across four domains, the total mental labor, and, then, the three dimensions: cognitive, managerial, and emotional labor. The mean score of total mental labor is 3.82.

What stands out most is the pattern in cognitive labor: the distribution among mothers is remarkably compressed and elevated, with relatively high minimum values. This suggests that nearly all mothers engage intensively in cognitive tasks, such as thinking about family needs. Mothers’ managerial and emotional labor levels, on the other hand, show slightly more variability.

**Figure 2:**
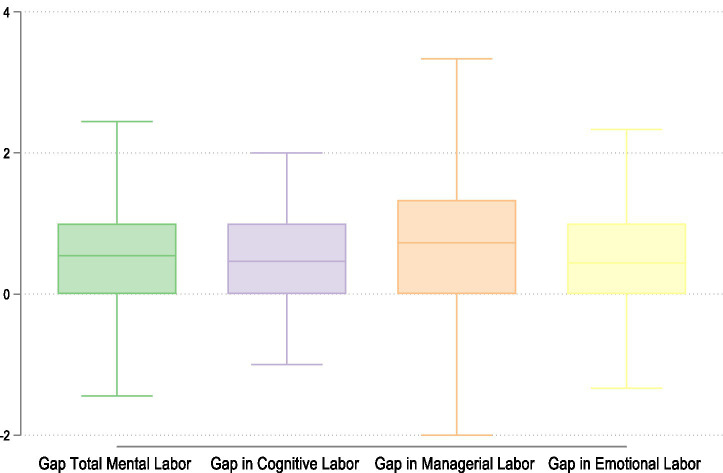
Perceived gap between individual and partner’s total mental labor, cognitive labor, managerial labor, and emotional labor. Source: Own elaborations on FORTIES data (2024).

Figure 2 illustrates the perceived gap in time dedicated to mental labor between mothers and their partners. Here we present how the gap is distributed also across the three dimensions, with positive values indicating that the mother perceives herself as doing more mental work than her partner, and negative values indicating the opposite.

On average, across all dimensions, the gap is positive, indicating that mothers perceive themselves as carrying a greater share of mental labor within the couple. The total mental labor gap shows that mothers report feeling that they dedicate more time to the invisible aspects of family life than their partners.

The managerial dimension exhibits the largest average gap. This suggests that mothers tend to perceive themselves as taking most of the responsibility for the logistical functioning of family life. Notably, managerial labor also shows the widest distribution, ranging from low negative to high positive values. This reflects both the unequal division and the heterogeneity in how couples negotiate this type of labor. Some mothers perceive more equity, while others report extreme imbalance. In contrast, cognitive labor shows a smaller mean gap and a more compressed distribution, suggesting more consistency in the perceived division of mental workload. Finally, emotional labor displays a similar average to cognitive labor, though with a slightly broader range.

**Figure 3:**
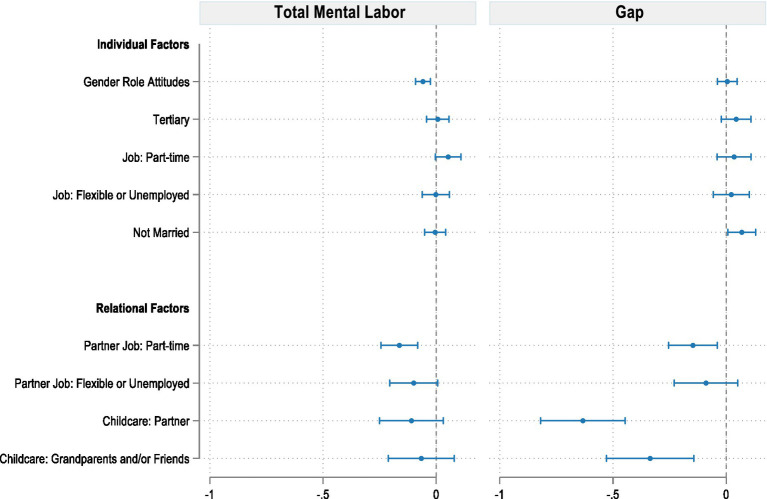
Regression models for labor and perceived gap in mental labor. Models control for geographic area of residence and type of recruitment. Source: Own elaborations on FORTIES data (2024). Full models are available in the Table A2 in the Appendix.

Figure 3 presents results from two regression models: the first one explores total maternal mental labor score (RQ1), while the second one models the perceived gap in mental labor between mothers and their partners (RQ2).

In the first model (left panel), two aspects show a significant negative association with total mental labor. More egalitarian gender role attitudes are linked to lower perceived time dedicated to mental activities. The effect size is modest: a 1-point increase in egalitarian attitudes on the 1–5 scale corresponds to a 0.059-point reduction in total mental labor. Similarly, having a partner who works part-time (vs. full-time) is associated with lower mental labor. Notably, help in childcare is not significant, as well as other dimensions related to mothers’ demographics or child characteristics.

The second model (right panel) sheds light on factors that shape the perceived mental labor gap within couples. Several variables are significantly associated with a smaller reported difference. When partners or grandparents actively engage in domestic child-rearing, mothers perceive a more equal distribution of mental labor. Effect sizes are relatively larger here than for individual characteristics; for example, partner help reduces the perceived gap by 0.633 points. Partners’ part-time employment and the child’s good or very good health are also associated with a smaller mental labor gap, likely due to reduced demands and more available support.

**Figure 4:**
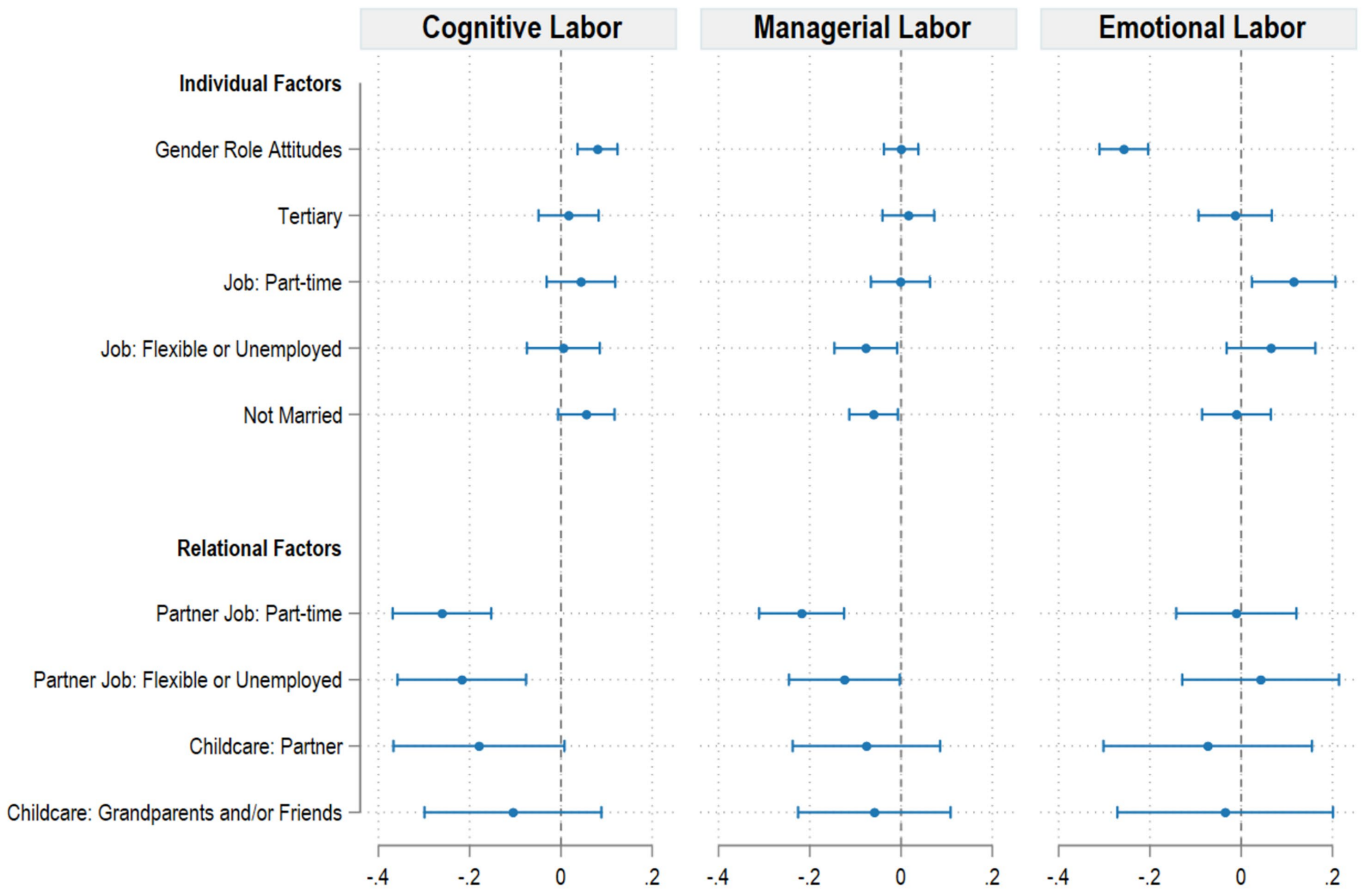
Regression models for cognitive, managerial, and emotional labor. Models control for geographic area of residence and type of recruitment. Source: Own elaborations on FORTIES data (2024). Full models are available in Table A3 in the Appendix.

To better understand how different dimensions of maternal mental labor are shaped by individual and relational aspects (RQ3), we ran three separate regression models using cognitive, managerial, and emotional labor as dependent variables. The results, shown in Figure 4, suggest that these three domains are shaped by partially distinct sets of predictors, supporting the idea that mental labor is a multidimensional construct. Some predictors are not significant in any of the models, such as the type of help in child-rearing (if any), mothers’ level of education and employment status, age, and marital status.

Among individual characteristics, in the emotional dimension, gender role attitudes are strongly and negatively associated with emotional labor: mothers with more egalitarian views report lower emotional burden. This is one of the strongest effects observed (*β* = −0.26, *p* < 0.001), suggesting that gender ideologies are particularly relevant when it comes to emotional burden within the family.

Emotional labor is also positively associated with mothers’ part-time employment: compared to full-time workers, those working part-time report higher emotional labor. This association may reflect a stronger emotional investment in the caregiving role among part-time working mothers, who may compensate for their reduced engagement in paid work by intensifying their involvement in the family sphere. From a relational perspective, spending more time at home could heighten both the opportunities and the perceived responsibility for managing others’ well-being.

At the same time, this pattern may also reflect broader socio-economic dynamics. Part-time employment is often more common among women with lower educational attainment and fewer career opportunities, who may experience greater emotional vulnerability and fewer resources to buffer the psychological costs of caregiving (Craig and Brown, 2017). These women might also have less access to external support, thereby internalizing a stronger sense of personal responsibility for emotional coordination within the household.

Finally, the child’s health status plays a crucial role. Mothers of children in very good health report significantly less emotional labor than those with children in fair or poor health, reinforcing the idea that emotional management is heightened in situations of vulnerability or concern.

The cognitive dimension shows a markedly different pattern. Interestingly, more egalitarian gender role attitudes are associated with higher cognitive labor. Although the coefficient is modest in magnitude (*β* = 0.08, *p* < 0.001), this effect is consistent and suggests that egalitarian women may engage more intensively in the cognitive aspects of family life. Cognitive labor is negatively associated with partners’ occupational status: when the partner works part-time or is flexible or unemployed, maternal cognitive labor decreases, presumably due to increased time spent by their partners around the house. These relational variables display comparatively larger effect sizes (e.g., *β* = −0.26 for partner part-time work), highlighting that time availability within couples exerts a stronger influence than individual attitudes on cognitive management.

For managerial labor, fewer variables reach statistical significance. However, some patterns emerge. Mothers working flexible hours or unemployed report slightly lower managerial labor, though the association is weak. The strongest predictor is again partners’ part-time work regime: when the partner is not employed full-time, maternal managerial labor decreases substantially. The effect size (*β* = −0.22, *p* < 0.001) is among the largest observed in this model, confirming that managerial responsibilities are particularly sensitive to the partner’s time flexibility and household presence. This supports the idea that managerial responsibilities can be more easily shared when the partner has greater time flexibility, allowing for the redistribution of household planning and coordination tasks.

Relational factors, particularly the partner’s employment status, show larger effect sizes for both cognitive (*β* = −0.26) and managerial (*β* = −0.22) labor, highlighting how shared availability and time allocation within the couple influence these dimensions more directly than individual characteristics.

## 5 Discussion

Mental labor is a multidimensional concept combining a strong individual component with a relational one, and manifesting in different spheres: cognitive, managerial, and emotional.

Concerning the first dichotomy, mental workload refers not only to the burden that falls on a single person, but also to an aspect inherently tied to relationships—to others for whom one thinks, plans, organizes, and worries. Our findings speak to both these directions: the individual and the relational. Both independent and dependent variables are, in fact, constructed to capture the individual and relational characteristics connected with mental labor.

Such a dual perspective allows us to observe how the two levels are deeply interconnected. Individual-level characteristics are more salient in explaining the overall intensity of mental labor. However, when analyzing the distribution and perceived imbalance of mental load within the couple, relational dynamics, especially help in childcare, become more relevant.

More in detail, we show that mothers dedicate substantial time to mental labor, and that this effort is particularly concentrated in the cognitive domain. When looking at overall mental labor, we find that individual characteristics such as gender role attitudes and the partner’s part-time work are key predictors. In line with theories of gender socialization and doing gender, more egalitarian attitudes are associated with a lower overall load for mothers, particularly in the managerial and emotional dimensions. In contrast, traditional attitudes reinforce women’s disproportionate responsibility. This finding is consistent with “gender socialization theory,” which emphasizes how early internalized norms predispose women to caregiving roles, and with the “doing gender” framework, which highlights how such expectations are continuously enacted and reinforced in daily practices. Similarly, when partners work part-time, mothers report a lower gap in mental labor, suggesting that time availability partially mitigates the inequality in its distribution. When focusing on the perceived gap between one’s own mental load and that attributed to the partner, relational factors become even more relevant.

The presence of support from the partner and from grandparents, as well as the partner’s part-time employment, is significantly associated with a narrower gap between perceived and attributed mental labor.

A closer look at the three dimensions of mental labor confirms this dual structure. Emotional labor is lower among mothers with more egalitarian gender role attitudes, likely reflecting a greater resistance to internalizing the emotional well-being of others as their sole responsibility, and confirming prior evidence that traditional gender norms tend to naturalize women’s emotional responsibility for others (Walzer, 1998; Wayne et al., 2023). At the same time, emotional labor increases among mothers working part-time, perhaps due to an intensification of caregiving roles or a stronger emotional investment in family life when more time is spent at home. Notably, emotional labor appears largely unaffected by partners’ time availability, reinforcing the idea that it is less redistributable and more deeply rooted in gendered expectations.

Cognitive labor, by contrast, shows a more complex pattern. Mothers with more egalitarian attitudes also report elevated levels of cognitive labor. On one hand, women with progressive beliefs may reject the moral obligation to be constantly emotionally available, thus reducing their emotional load. On the other hand, they may still internalize the responsibility for coordinating family life in response to persistent gendered expectations. From this perspective, cognitive labor becomes a subtle form of doing gender (West and Zimmerman, 1987), even in households where formal equality is aspired to. An additional possible explanation is that cognitive tasks, such as anticipating needs or tracking logistics, are more automatic and less consciously recognized than other forms of domestic work, making them harder to delegate or resist, even for those who actively seek a more equal division of labor. Furthermore, this pattern may also align with intensive parenting norms (Chaloupková, 2025). Recent evidence shows that intensive parenting behaviors are often associated with egalitarian gender beliefs and a rejection of gender essentialism (Lankes, 2022). Our findings, moreover, may reflect the fact that greater equality in the division of domestic tasks often entails more negotiation and coordination between partners. As highlighted by Bittman et al. (2003), household labor is the outcome of ongoing bargaining processes, and such negotiation itself requires monitoring, planning, and cognitive effort, which may add to women’s mental load even in couples who perceive themselves as egalitarian. This result resonates with the time availability approach, insofar as coordination and bargaining needs persist even when domestic work is more equally shared.

Managerial labor follows a similar pattern to the cognitive dimension: it is significantly lower when the partner works part-time, suggesting that time availability facilitates the redistribution of organizational tasks. However, emotional labor seems largely insensitive to this structural factor, reinforcing the idea that emotional responsibility is more deeply rooted in identity and less easily externalized.

Taken together, these findings illustrate that the three theoretical perspectives—gender socialization, doing gender, and time availability—help to capture complementary but distinct mechanisms of mental labor. Gender norms shape the unequal internalization of responsibilities, everyday practices reproduce women’s role as default managers, and structural constraints in time allocation limit redistributive possibilities. Our evidence suggests that only by considering these mechanisms jointly can we fully explain the persistence of unequal mental labor.

These patterns are not entirely surprising in the Italian context, where deeply rooted traditional norms and persistent gender inequalities continue to shape the organization of care (Mussida and Patimo, 2021). In such a setting, reducing the mental burden on mothers—and narrowing the perceived gap between partners—may require not only changes in time availability or external support, but also a more profound cultural shift in how responsibility is shared, anticipated, and emotionally internalized.

Future research could extend this approach to other European countries to assess the extent to which similar patterns hold in different institutional and cultural settings. Such cross-country analyses would also shed light on how work–family policies and normative frameworks interact in shaping the distribution of mental labor, helping to disentangle what is context-specific from what is more general. At the same time, the divergence from the original 9-item scale by Wayne et al. (2023) reduces comparability with studies using the full instrument, which we acknowledge as a limitation. Finally, our findings raise important policy implications: on the one hand, work–family policies such as childcare provision and incentives for fathers’ leave can directly alleviate women’s overload; on the other, normative policy feedback effects may gradually reshape gender expectations, making more balanced arrangements socially legitimate. Both aspects appear crucial to address this enduring form of inequality.

## 6 Conclusion

This study provides new empirical evidence on the multidimensional nature of maternal mental labor and its unequal distribution within Italian families. Drawing on data from more than two thousand mothers, it shows that mental labor—although invisible and intangible—constitutes a pervasive component of family management, combining cognitive, managerial, and emotional efforts that are deeply gendered. Italian mothers report high levels of invisible mental tasks, particularly in the cognitive and managerial dimensions, and perceive a marked imbalance relative to their partners. Mothers report high levels of invisible mental tasks—particularly cognitive and managerial—and perceive a marked imbalance relative to their partners.

The findings highlight how gender norms, relational dynamics, and structural constraints intersect in shaping the distribution of mental labor, revealing that emotional labor, in particular, remains resistant to redistribution. Addressing this persistent inequality requires coordinated efforts across both policy and cultural domains. While work–family policies may alleviate part of the burden, achieving a more equitable distribution of mental labor ultimately hinges on a fundamental redefinition of caregiving roles and gendered expectations within heterosexual family formations.

Ultimately, this study underlines that achieving gender equality in the family sphere cannot rely solely on redistributing physical tasks. The invisible dimensions of planning, worrying, and coordinating remain the last frontier of domestic inequality. Recognizing and making visible this mental labor is a necessary step toward a more equitable sharing of care and toward improving the well-being of both women and families as a whole.

## Data Availability

The dataset presented in this article is not currently publicly available due to data-sharing restrictions agreed with the funding agency. In accordance with these agreements, an anonymized version of the dataset will be made publicly available three years after the end of the project, which is scheduled for 2026.

## Footnotes

1 The full list of items is available in Table A1 in the Appendix.
