# Supplementary material for: Understanding the dimensions of mental labor: the invisible load of Italian mothers
Source: Front Sociol. 2026 Jan 29;10:1683261. doi: 10.3389/fsoc.2025.1683261 (PMC12893982; doi:10.3389/fsoc.2025.1683261)
Supplement: Supplementary file 1 [file Table_1.docx]

**Appendix**

*Tab. A1 Mental Labor Items*

| **How often do you**  *(1 Never – 5 Always)* |  |
| --- | --- |
| Think about your family’s needs, goals, activities, responsibilities, and/or well-being | Cognitive Labor |
| Make arrangements for what needs to get done for your family’s needs, goals, activities, responsibilities,  and/or well-being | Managerial Labor |
| Coordinate your family’s needs, goals, activities, responsibilities, and/or well-being |  |
| Make plans for your family’s needs, goals, activities, responsibilities, and/or well-being |  |
| Feel apprehensive about your family’s needs, goals, activities, responsibilities, and/or well-being | Emotional Labor |
| Feel troubled by your family’s needs, goals, activities, responsibilities, and/or well-being |  |
| Feel distressed about your family’s needs, goals, activities, responsibilities, and/or well-being |  |

*Tab. A2. Regression Models: Total Mental Labor (1) and Gap in Mental Labor (2) among mothers of one child. Full Specifications. OLS estimates with robust standard errors. All models include controls for maternal age, child age and health, geographical area, and recruitment channel. Reference categories are indicated in parentheses.*

| VARIABLES | | Total Mental Labor  (1) | Gap in Mental Labor  (2) |
| --- | --- | --- | --- |
| **Individual Factors** |  |  |  |
|  | Gender Role Attitudes | -0.059*** | 0.004 |
| *Education (ref: Lower than Tertiary)* |  | (0.020) | (0.026) |
|  | Tertiary | 0.006 | 0.043 |
|  |  | (0.030) | (0.039) |
| *Occupation (ref: Full-time)* |  |  |  |
|  | Part- Time | 0.052 | 0.034 |
|  |  | (0.034) | (0.045) |
|  | Flexible or Unemployed | -0.002 | 0.022 |
|  |  | (0.036) | (0.048) |
| *Marital Status(ref:Married)* |  |  |  |
|  | Not Married | -0.004 | 0.068* |
|  |  | (0.028) | (0.037) |
| **Relational Factors** |  |  |  |
| *Partner Occupation(ref: Full-time)* |  |  |  |
|  | Part-time | -0.163*** | -0.147** |
|  |  | (0.049) | (0.065) |
|  | Flexible or Unemployed | -0.099 | -0.089 |
|  |  | (0.064) | (0.089) |
| *Help in Childcare(ref: Nobody)* |  |  |  |
|  | Partner | -0.110 | -0.633*** |
|  |  | (0.085) | (0.116) |
|  | Grandparents and/or Friends | -0.066 | -0.336*** |
|  |  | (0.085) | (0.120) |
| **Basic Controls** |  |  |  |
|  | Mother’s Age | 0.009*** | 0.014*** |
|  |  | (0.002) | (0.003) |
|  | Age of First Child | -0.001 | 0.005 |
|  |  | (0.005) | (0.007) |
| *First Child Health(ref: Very Good)* |  |  |  |
|  | Bad | 0.034 | 0.205*** |
|  |  | (0.048) | (0.063) |
|  | Good | -0.019 | -0.023 |
|  |  | (0.032) | (0.043) |
| *Geographical Area(ref: North West)* |  |  |  |
|  | North East | 0.012 | 0.035 |
|  |  | (0.038) | (0.051) |
|  | Central | -0.017 | -0.053 |
|  |  | (0.040) | (0.053) |
|  | South and Islands | 0.066* | -0.119** |
|  |  | (0.037) | (0.050) |
| *Recruitment(ref: Panel)* |  |  |  |
|  | Social | 0.151*** | 0.231*** |
|  |  | (0.034) | (0.045) |
|  | Constant | 3.705*** | 0.384** |
|  |  | (0.150) | (0.198) |
|  |  |  |  |
|  | Observations | 2,309 | 2,309 |
|  | R-squared | 0.034 | 0.089 |
|  | Adj R-squared | 0.026 | 0.082 |
|  | Mean VIF | 2.01 | 2.01 |

*Tab. A3. Regression Models: Cognitive Labor (1), Managerial Labor (2), and Emotional Labor (3) among mothers of one child. Full Specifications. OLS estimates with robust standard errors. All models include controls for maternal age, child age and health, geographical area, and recruitment channel. Reference categories are indicated in parentheses.*

| VARIABLES | | Cognitive Labor  (1) | Managerial Labor  (2) | Emotional Labor  (3) |
| --- | --- | --- | --- | --- |
| **Individual Factors** |  |  |  |  |
|  | Gender Role Attitudes | 0.0802*** | 0.000119 | -0.257*** |
| *Education (ref: Lower than Tertiary)* |  | (0.0266) | (0.0230) | (0.0325) |
|  | Tertiary | 0.0168 | 0.0161 | -0.0131 |
|  |  | (0.0400) | (0.0345) | (0.0488) |
| *Occupation (ref: Full-time)* |  |  |  |  |
|  | Part- Time | 0.0439 | -0.00121 | 0.115** |
|  |  | (0.0457) | (0.0394) | (0.0557) |
|  | Flexible or Unemployed | 0.00535 | -0.0773* | 0.0654 |
|  |  | (0.0485) | (0.0418) | (0.0591) |
| *Marital Status(ref:Married)* |  |  |  |  |
|  | Not Married | 0.0557 | -0.0602* | -0.0104 |
|  |  | (0.0376) | (0.0324) | (0.0458) |
| **Relational Factors** |  |  |  |  |
| *Partner Occupation(ref: Full-time)* |  |  |  |  |
|  | Part-time | -0.261*** | -0.218*** | -0.0107 |
|  |  | (0.0656) | (0.0566) | (0.0800) |
|  | Flexible or Unemployed | -0.217** | -0.124* | 0.0426 |
|  |  | (0.0856) | (0.0738) | (0.104) |
| *Help in Childcare(ref: Nobody)* |  |  |  |  |
|  | Partner | -0.180 | -0.0759 | -0.0733 |
|  |  | (0.114) | (0.0982) | (0.139) |
|  | Grandparents and/or Friends | -0.105 | -0.0585 | -0.0351 |
|  |  | (0.118) | (0.102) | (0.144) |
| **Basic Controls** |  |  |  |  |
|  | Mother’s Age | 0.0174*** | 0.00523* | 0.00605 |
|  |  | (0.00354) | (0.00305) | (0.00431) |
|  | Age of First Child | -0.0109 | 0.00804 | -0.000982 |
|  |  | (0.00794) | (0.00685) | (0.00968) |
| *First Child Health(ref: Very Good)* |  |  |  |  |
|  | Bad | -0.118* | -0.0963* | 0.316*** |
|  |  | (0.0641) | (0.0552) | (0.0781) |
|  | Good | -0.0976** | -0.148*** | 0.186*** |
|  |  | (0.0434) | (0.0374) | (0.0529) |
| *Geographical Area(ref: North West)* |  |  |  |  |
|  | North East | 0.0579 | 0.00815 | -0.0278 |
|  |  | (0.0516) | (0.0445) | (0.0629) |
|  | Central | -0.0368 | -0.0313 | 0.0164 |
|  |  | (0.0534) | (0.0460) | (0.0651) |
|  | South and Islands | 0.0423 | 0.0146 | 0.141** |
|  |  | (0.0502) | (0.0433) | (0.0612) |
| *Recruitment(ref: Panel)* |  |  |  |  |
|  | Social | 0.302*** | 0.129*** | 0.0233 |
|  |  | (0.0460) | (0.0396) | (0.0560) |
|  | Constant | 3.410*** | 3.862*** | 3.842*** |
|  |  | (0.199) | (0.172) | (0.243) |
|  |  |  |  |  |
|  | Observations | 2,309 | 2,309 | 2,309 |
|  | R-squared | 0.099 | 0.035 | 0.054 |
|  | Adj. R-squared | 0.092 | 0.028 | 0.046 |
|  | Mean VIF | 2.01 | 2.01 | 2.01 |

*Tab. A4. Regression Models: Total Mental Labor (1), Gap in Mental Labor (2), Cognitive Labor (3), Managerial Labor (4), and Emotional Labor (5) on the full sample.. Full Specifications. OLS estimates with robust standard errors. All models include controls for maternal age, child age and health, geographical area, and recruitment channel. Reference categories are indicated in parentheses.*

| VARIABLES | | Total Mental Labor  (1) | Gap  (2) | Cognitive Labor  (3) | Managerial Labor  (4) | Emotional Labor  (5) |
| --- | --- | --- | --- | --- | --- | --- |
| **Individual Factors** |  |  |  |  |  |  |
|  | Gender Role Attitudes | -0.053*** | 0.017 | 0.087*** | 0.006 | -0.254*** |
|  |  | (0.017) | (0.023) | (0.023) | (0.020) | (0.028) |
| *Education (ref: Lower than Tertiary)* |  |  |  |  |  |  |
|  | Tertiary | 0.015 | 0.038 | 0.011 | 0.031 | 0.003 |
|  |  | (0.026) | (0.035) | (0.035) | (0.030) | (0.042) |
| *Occupation (ref: Full-time)* |  |  |  |  |  |  |
|  | Part- Time | 0.035 | 0.023 | 0.025 | -0.033 | 0.113** |
|  |  | (0.030) | (0.040) | (0.040) | (0.035) | (0.049) |
|  | Flexible or Unemployed | -0.022 | 0.004 | 0.001 | -0.073** | 0.003 |
|  |  | (0.032) | (0.042) | (0.042) | (0.037) | (0.051) |
| *Marital Status(ref:Married)* |  |  |  |  |  |  |
|  | Not Married | -0.000 | 0.054* | 0.062* | -0.057** | -0.007 |
|  |  | (0.025) | (0.033) | (0.033) | (0.029) | (0.040) |
| **Relational Factors** |  |  |  |  |  |  |
| *Partner Occupation(ref: Full-time)* |  |  |  |  |  |  |
|  | Part-time | -0.129*** | -0.096* | -0.208*** | -0.192*** | 0.013 |
|  |  | (0.044) | (0.058) | (0.058) | (0.051) | (0.071) |
|  | Flexible or Unemployed | -0.098* | -0.087 | -0.229*** | -0.138** | 0.071 |
|  |  | (0.054) | (0.072) | (0.073) | (0.063) | (0.088) |
| *Help in Childcare(ref: Nobody)* |  |  |  |  |  |  |
|  | Partner | -0.138* | -0.614*** | -0.121 | -0.035 | -0.257** |
|  |  | (0.070) | (0.093) | (0.094) | (0.082) | (0.114) |
|  | Grandparents and/or Friends | -0.068 | -0.300*** | -0.065 | 0.004 | -0.146 |
|  |  | (0.073) | (0.096) | (0.098) | (0.085) | (0.118) |
| **Basic Controls** |  |  |  |  |  |  |
|  | Mother’s Age | 0.008*** | 0.014*** | 0.017*** | 0.005* | 0.002 |
|  |  | (0.002) | (0.003) | (0.003) | (0.002) | (0.003) |
|  | Age of First Child | -0.000 | 0.008 | -0.009 | 0.006 | 0.002 |
|  |  | (0.004) | (0.006) | (0.006) | (0.005) | (0.007) |
| *First Child Health(ref: Very Good)* |  |  |  |  |  |  |
|  | Bad | 0.021 | 0.157*** | -0.114** | -0.100** | 0.277*** |
|  |  | (0.042) | (0.056) | (0.056) | (0.049) | (0.068) |
|  | Good | 0.010 | -0.016 | -0.061 | -0.108*** | 0.201*** |
|  |  | (0.028) | (0.037) | (0.038) | (0.033) | (0.045) |
| *Geographical Area(ref: North West)* |  |  |  |  |  |  |
|  | North East | 0.004 | 0.005 | 0.030 | 0.004 | -0.020 |
|  |  | (0.034) | (0.045) | (0.046) | (0.040) | (0.055) |
|  | Central | -0.025 | -0.063 | -0.046 | -0.024 | -0.003 |
|  |  | (0.035) | (0.046) | (0.047) | (0.041) | (0.057) |
|  | South and Islands | 0.039 | -0.149*** | -0.007 | 0.000 | 0.126** |
|  |  | (0.033) | (0.043) | (0.044) | (0.038) | (0.053) |
| *Recruitment(ref: Panel)* |  |  |  |  |  |  |
|  | Social | 0.158*** | 0.230*** | 0.304*** | 0.140*** | 0.030 |
|  |  | (0.031) | (0.040) | (0.040) | (0.035) | (0.049) |
| *Parity* |  |  |  |  |  |  |
|  | Two Children | 0.022 | 0.082* | 0.095** | 0.032 | -0.061 |
|  |  | (0.031) | (0.042) | (0.042) | (0.037) | (0.051) |
|  | Constant | 3.745*** | 0.335** | 3.356*** | 3.778*** | 4.102*** |
|  |  | (0.129) | (0.170) | (0.172) | (0.150) | (0.208) |
|  |  |  |  |  |  |  |
|  | Observations | 2,988 | 2,988 | 2,988 | 2,988 | 2,988 |
|  | R-squared | 0.033 | 0.093 | 0.096 | 0.035 | 0.054 |
|  | Adj. R-squared | 0.026 | 0.087 | 0.091 | 0.029 | 0.048 |
|  | Mean VIF | 1.88 | 1.88 | 1.88 | 1.88 | 1.88 |
